# Characterization of Microbiota that Influence Immunomodulatory Effects of Fermented *Brassica rapa* L

**DOI:** 10.1264/jsme2.ME19003

**Published:** 2019-06-06

**Authors:** Bayanjargal Sandagdorj, Chisato Hamajima, Takeshi Kawahara, Jun Watanabe, Sachi Tanaka

**Affiliations:** 1 Food Research Institute, National Agriculture and Food Research Organization Tsukuba, Ibaraki 305–8642 Japan; 2 School of Integrative and Global Majors, University of Tsukuba Tsukuba, 305–8577 Japan; 3 Academic Assembly (Institute of Agriculture), Shinshu University Minamiminowa, Nagano 399–4598 Japan; 4 Supramolecular Complexes Unit, Research Center for Fungal and Microbial Dynamism, Shinshu University Minamiminowa, Nagano 399–4598 Japan

**Keywords:** lactic acid bacteria, *Brassica rapa* L., fermentation, IFN-γ, IL-10

## Abstract

Lactic acid bacteria (LAB) exert beneficial health effects by regulating immune responses. *Brassica rapa* L., known as Nozawana, is commonly consumed as a lactic acid-fermented food called nozawana-zuke. Few studies have investigated changes in the bacterial community and cytokine production activities during the fermentation of *B. rapa* L. In order to obtain more detail information, we herein conducted a study on fresh *B. rapa* L. fermented for 28 d. An amplicon analysis of the 16S rRNA gene revealed that *Lactobacillales* predominated during fermentation, and the microbiota became less diverse on day 7 or later. Fermented *B. rapa* L. promoted the production of interferon (IFN)-γ and interleukin (IL)-10 by mouse spleen cells more than non-fermented vegetables. *Lactobacillus curvatus* was the predominant species during fermentation, followed by *L. plantarum* and *L. brevis*. *L. sakei* was occasionally detected. A correlation analysis showed that IFN-γ concentrations positively correlated with the numbers of *L. curvatus* and *L. plantarum*, while those of IL-10 correlated with the numbers of *L. sakei* in addition to these 2 species. Significantly higher levels of IFN-γ and IL-10 were induced by fermented *B. rapa* L. when isolated *Lactobacillus* strains were added as starter cultures. These results suggest that the *Lactobacillus* species present in fermented *B. rapa* L. are beneficial for manufacturing vegetables with immunomodulatory effects.

Lactic acid bacteria (LAB) are widely distributed in nature and are used to produce fermented foods. LAB exert beneficial health effects including the regulation of immune responses. In animal models, LAB supplementation prevents chemically induced colitis, asthma, and allergic rhinitis by down-regulating inflammatory cytokine production or inducing anti-inflammatory cytokine production (4).

T cells and natural killer (NK) cells produce the cytokine interferon (IFN)-γ, which activates dendritic cells (DCs) and macrophages to fight against infections (3). *Lactobacillus pentosus* strain S-PT84 induces interleukin (IL)-12 production by activating the Toll-like receptor (TLR) isoforms TLR2, TLR4, or both, in DCs (20). Furthermore, S-PT84 induces IFN-γ production by NK1.1^+^ cells in an IL-12-dependent manner (20).

DCs, macrophages, and regulatory T (Treg) cells produce the anti-inflammatory cytokine IL-10. This cytokine inhibits the activation of macrophages, T cells, and NK cells and suppresses the production of proinflammatory cytokines (8). Enhancements in IL-10 production have been shown to contribute to the anti-inflammatory effects of certain LAB strains. For example, *L. plantarum* strains stimulate IL-10 production by macrophages, whereas *L. reuteri* and *L. casei* strains induce IL-10-producing Treg cells by modulating the functions of DCs (23, 31).

*L. sakei* and *L. plantarum* strains derived from kimchi differ in their cytokine production patterns and regulatory effects on T helper (Th)1/Th2-mediated immune responses (12). For example, *L. plantarum* strain YU, which is present in fermented food, inhibits viral infections by enhancing the production of IL-12 and IFN-γ by immune cells (16). Allergic inflammation is characterized by the infiltration of tissues by mast cells and activated eosinophils, which release Th2 cytokines, particularly IL-4 and IL-5 (24). IL-12 and IFN-γ suppress Th2 differentiation, and IL-10 is a potent inhibitor of inflammation through its suppression of the production of Th2 cytokines (7). The *L. pentosus* strain S-PT84 induces the production of IL-12 and IL-10 *in vitro*. In a mouse model of OVA-induced allergy, orally administered S-PT84 decreased the concentration of serum IgE and inhibited the active cutaneous anaphylaxis reaction as well as the production of IL-4 by spleen cells (25). Moreover, IL-10 production by the splenocytes of OVA-immunized mice was found to be increased by dietary S-PT84 (25). Therefore, LAB that induce the production of IL-12, IFN-γ, and IL-10 may exert preventive and therapeutic effects for treating allergies.

*Brassica rapa* L., known as Nozawana, is a traditional vegetable in Japan. In the Nagano area of Japan, *B. rapa* L. is often consumed as a lactic acid-fermented food called nozawana-zuke. We previously reported that *B. rapa* L. enhanced natural killer activity and IFN-γ production by mouse spleen cells through an IL-12-dependent mechanism (37). We also demonstrated that fresh and fermented *B. rapa* L. induced changes in short-chain fatty acid production in the colon and cecum of mice, which induced immunoregulatory effects (33, 34). Furthermore, *L. fermentum* isolated from nozawana-zuke enhanced IFN-γ and IL-12p40 mRNA expression in mouse spleen cells (15). Thus, increased numbers of LAB during the fermentation of *B. rapa* L. may activate immune cells to produce cytokines. However, few studies have investigated changes in the bacterial community and cytokine production during the fermentation of *B. rapa* L.

To address this insufficiency in our knowledge with the aim of enhancing the beneficial effects of fermented *B. rapa L*., we herein performed an amplicon analysis of the 16S rRNA gene to analyze the bacterial community during the fermentation of *B. rapa* L. and identify LAB species involved in the induction of cytokines by fermented *B. rapa* L. We also isolated LAB strains from fermented *B. rapa* L. for use as starter cultures to increase the production of cytokines.

## Materials and Methods

### Preparation of fermented *B. rapa* L

Fresh *B. rapa* L. (approximately 5 kg), purchased from Takeuchi Nousan (Nagano, Japan), was washed with tap water and then fermented in 20-L pickle jars containing a salt solution (7% w/w, NaCl) at 10°C for 28 d. Vegetables (approximately 500 g each) were collected on days 0, 3, 7, 14, 21, and 28 after the start of fermentation. Three independent experiments were conducted using different plant materials to prepare fermented *B. rapa* L.

Fresh or fermented *B. rapa* L. was suspended in phosphate-buffered saline (PBS), and the suspension was passed through a 100-μm nylon cell strainer (BD Biosciences, San Jose, CA, USA) to eliminate large particles. Filtrates were centrifuged at 20,630×*g* for 5 min, and the pellets obtained were used as the LAB suspension (LS). LS was treated with RNAlater (Qiagen, Hilden, Germany) and stored at 4°C. In the immunological analysis, LS was heated at 65°C for 30 min to kill bacteria and then lyophilized (FDU-1200 freeze dryer; Eyela, Tokyo, Japan). Lyophilized LS was stored at −30°C.

### Isolation of LAB

LAB were isolated from *B. rapa* L. fermented for 28 d according to a modified procedure (15). Briefly, a sample was diluted with PBS and plated on de Man, Rogosa, Sharpe (MRS) agar (Kanto Chemical, Tokyo, Japan) containing 0.5% (w/v) CaCO_3_. After an anaerobic incubation at 29°C for 48 h, the colonies surrounded with clear CaCO_3_-dissolution zones were transferred to MRS broth (Difco Laboratories, Detroit, MI, USA). The isolated strains were suspended in 10% glycerol and stored at −80°C. In the immunological analysis, 46 strains were cultured at 29°C for 48 h in MRS broth. Cells were harvested by centrifugation at 20,630×*g* for 15 min, washed with MilliQ water, and heated at 65°C for 30 min. Heat-killed cells were lyophilized and stored at −30°C.

### DNA extraction

Bacterial DNA extracted from LS or isolated bacterial strains was purified using a QIAamp DNA Stool Mini Kit (Qiagen) and ZircoPrep Mini Kit (Nippon Genetics, Tokyo, Japan) according to the manufacturers’ instructions.

### 16S rRNA gene amplicon analysis

DNA samples were quantified, followed by the PCR amplification of the V3 and V4 regions of 16S rRNA genes. PCR primers were 341F (5ʹ-CCTACGGGNGGCWGCAG-3ʹ) (19) and 806R (5ʹ-GGACTA CHVGGGTWTCTAAT-3ʹ) (17) joined to the Illumina overhang adapter sequences. Second PCR was performed to add barcodes to each sample. After quantification, amplicons were pooled in equal amounts, and pair-end 2×300 bp sequencing was performed using a MiSeq System (Illumina, San Diego, CA, USA) and MiSeq Reagent Kit v3 (Illumina).

Sequences in the demultiplexed format were analyzed using QIIME 1.9.0 (5). Merged paired-end reads were quality-filtered with default settings. The filtered sequences were then clustered into operational taxonomic units (OTUs) using Greengenes 13.8 as reference sequences for taxonomy assignments at 97% identity (22). A representative sequence of each OTU was selected, and chimeric sequences were identified using VSEARCH (28), with singletons filtered from the OTU table. OTUs assigned as originating from chloroplast sequences were eliminated from further analyses. Alpha diversity was analyzed by rarefying the OTU table at a consistent sampling depth of 34,100 sequences.

Phylogenetic metrics were compared by estimating Faith’s Phylogenetic Diversity (PD_whole_tree) between samples, and species richness was estimated using Chao1, the Shannon index, and number of OTUs (9). Representative sequences of major OTUs were compared using the BLAST algorithm to query a public-domain database (1). Ward’s hierarchical clustering method using a weighted Unifrac distance matrix was performed using the APE package of R 3.4.4 (27).

### Real-time quantitative PCR analysis of *Lactobacillus* species and the total *Lactobacillus* population

*L. curvatus*, *L. plantarum*, *L. brevis*, *L. sakei*, and total lactobacilli present in LS preparations were quantified using real-time quantitative PCR with primer pairs specific to each *Lactobacillus* species and the genus *Lactobacillus* (2, 10, 26, 35). PCR reaction mixtures (25 μL) contained 12.5 μL KAPA SYBR Fast qPCR Master Mix (KAPA Biosystems, Wilmington, MA, USA), 100 pmol each of forward and reverse primers, and 1 μL of DNA. Reaction conditions were 95°C for 30 s, followed by 40 cycles at 95°C for 10 s, and 60°C for 30 s. Fluorescent products were detected at the last step of each cycle. A melting curve analysis was performed after amplification to identify target amplicons. A standard curve for each *Lactobacillus* species was generated using the genomic DNA of the type-strain cultures JCM 1059, 1096, 1149, and 1157. All samples and standard genomic DNAs were analyzed in duplicate.

### Identification of isolated bacterial strains

LAB strains isolated from fermented *B. rapa* L. were identified according to their 16S rRNA gene sequences and PCR amplification using the species-specific primers shown above. The 16S rRNA gene was amplified using PCR with the primers 27F and 1492R (18). PCR products were separated on 1.5% agarose gels, purified, and used as templates for sequencing reactions with the primer 926R (30). Sequences were read using an ABI Prism 310 Genetic Analyzer (Thermo Fisher Scientific Japan, Tokyo, Japan).

### Preparation of starter cultures

*L. plantarum* strain Lp4 and *L. curvatus* strain Lc3 were grown in MRS at 29°C for 24 h. Cells were washed with saline and then suspended in saline. Cell suspensions (1×10^10^ cfu) were inoculated into 1 kg of fresh *B. rapa* L. in a salt solution (7% w/w, NaCl) in a pickle jar at 10°C for 3 d. Naturally fermented *B. rapa* L. (NF) was prepared in a similar manner except for the addition of starter cultures. Samples were obtained 3 d after the start of fermentation. LS was prepared from each sample following the procedures described above.

### Preparation and culture of mouse spleen cells

Male C57BL/6 mice (CLEA Japan, Tokyo, Japan) were housed at 23±2°C with a 12-h light/dark cycle. All experimental procedures were conducted in accordance with the Regulations for Animal Experimentation of Shinshu University, Japan. The preparation of spleen cells involved a treatment with 0.17 M Tris-HCl buffer (pH 7.65) containing 0.83% NH_4_Cl to deplete red blood cells. After centrifugation, cells were resuspended in RPMI-1640 medium containing 10% (v/v) fetal calf serum, 100 U mL^–1^ penicillin G, and 100 μg mL^–1^ streptomycin. Cells (5×10^5^ cells per well) were cultured in 96-well flat-bottomed plates in the presence of LS or LAB at 37°C for 48 h in an atmosphere containing 5% CO_2_.

### Enzyme-linked immunosorbent assay (ELISA)

The concentrations of IFN-γ and IL-10 in culture supernatants were measured using ELISA kits (eBioscience, San Diego, CA, USA) according to the manufacturer’s instructions.

### Statistical analysis

Data represent the mean±standard deviation (SD). Statistical analyses were performed using a one-way ANOVA followed by Tukey’s test for group comparisons. *P*<0.05 was considered to be significant. The relationship between cytokine levels and the number of 16S rRNA genes of each *Lactobacillus* species was analyzed using Pearson’s correlation analysis. GraphPad Prism (GraphPad Software, San Diego, CA, USA) was used for data analysis.

## Results

### Changes in the microbial community during the fermentation of *B. rapa* L

High-quality 16S rRNA gene sequences (*n*=968,180) were analyzed (average, 53,788±22,818 sequences per sample). After excluding chimeric sequences or singletons from the OTU table, sequences were divided into 2,979 OTUs. We further excluded 428 OTUs that were identified as chloroplast sequences.

**Fig. 1:**
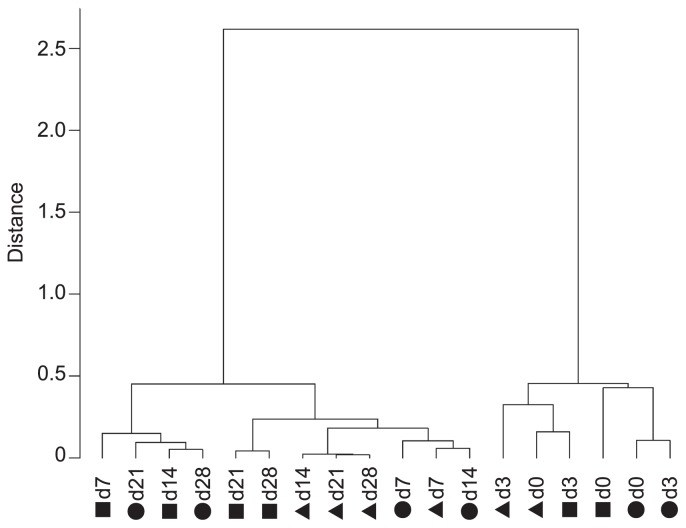
Hierarchical clustering of microbiotas of fermented and non-fermented *B. rapa* L. *B. rapa* L. fermentation was conducted at 10°C for 28 d, and samples were acquired 0, 3, 7, 14, 21, and 28 d after the start of fermentation. The V3–V4 regions of the 16S rRNA gene were amplified, and an amplicon analysis was performed using a MiSeq System. Amplicon analysis data were analyzed with the QIIME pipeline, and hierarchical clustering of the microbial population of each sample was performed using a weighted Unifrac distance matrix. Circles, triangles, and squares represent different batches of fermented *B. rapa* L. preparations.

Before and on day 3 of fermentation, most bacteria were identified as *Rhizobiales*, *Sphingomonadales*, and *Pseudomonadales*. The relative abundance of these bacteria significantly decreased on day 7 or 14 (Table 1). In contrast, *Lactobacillales* became the most predominant order on day 7 or later, reaching a relative abundance of ≥70% (Table 1). All alpha diversity parameters were significantly lower on day 7 than those of non-fermented vegetables (Table 2). The hierarchical cluster analysis using a weighted Unifrac distance matrix indicated that the microbiotas of non-fermented and fermented *B. rapa* L. comprised two main clusters (Fig. 1). One of the main clusters consisted of the microbiotas of non-fermented and 3-d fermented *B. rapa* L., and the other comprised those of vegetables fermented for ≥7 d.

*Lactobacillaceae* was the most dominant family among the bacteria assigned to the order *Lactobacillales*, and relative abundance exceeded 70% on day 14. Among the family *Lactobacillaceae*, representative sequences of the four most dominant OTUs showed the highest sequence identities with the 16S rRNA genes of *L. curvatus*, *L. plantarum*, *L. sakei*, and *L. brevis*.

### Changes in the number of *Lactobacillus* species during the fermentation of *B. rapa* L

**Fig. 2:**
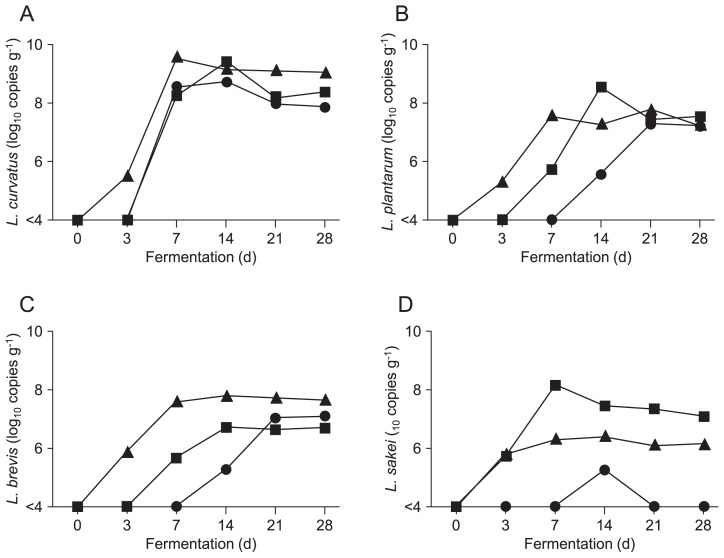
Changes in the LAB population during the fermentation of *B. rapa* L. Fermentation was performed as described in Fig. 1. We used real-time PCR to quantify *L. curvatus* (A), *L. plantarum* (B), *L. brevis* (C) and *L. sakei* (D) in DNA samples of LS. A standard curve for each *Lactobacillus* species was generated using the genomic DNA of type strain cultures. Data from three independent experiments are shown. Circles, triangles, and squares represent different batches of fermented *B. rapa* L. preparations.

Although *L. curvatus*, *L. plantarum*, *L. brevis*, and *L. sakei* were not detected in fresh *B. rapa* L., they were present in all batches of fermented *B. rapa* L. (Fig. 2). Among them, *L. curvatus* (Fig. 2A) was the most numerous lactobacillus throughout fermentation, reaching a maximum on day 7. *L. plantarum* (Fig. 2B) and *L. brevis* (Fig. 2C) were the second most populous *Lactobacillus* species. In contrast, the number of *L. sakei* (Fig. 2D) was markedly lower than those of other lactobacilli and varied among the batches. The populations of these *Lactobacillus* species became relatively stable on day 14 or later.

### Changes in cytokine production induced by fermented *B. rapa* L

**Fig. 3:**
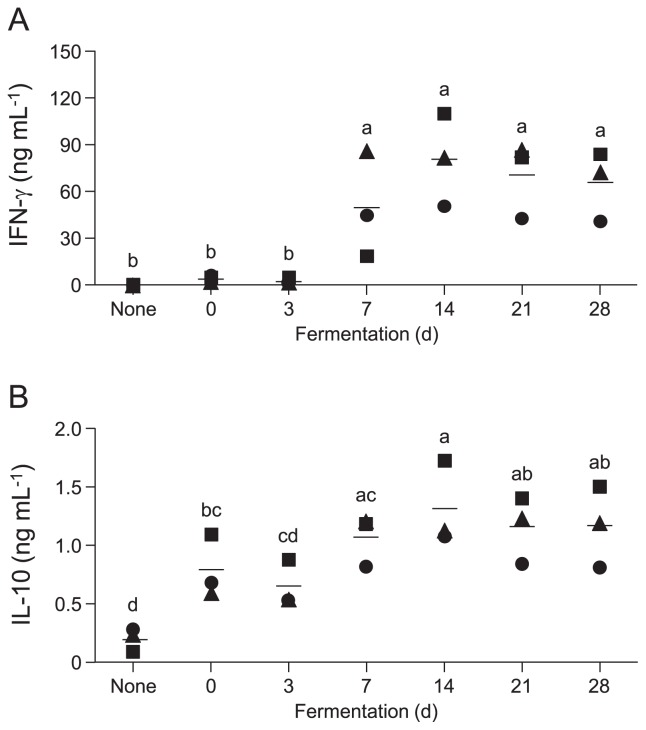
Changes in cytokine induction activity of fermented *B. rapa* L. *B. rapa* L. fermentation was performed as described in Fig. 1. Mouse spleen cells (5×10^5^ cells per well) were treated with 0.25 and 10 μg mL^–1^ of LS to measure IFN-γ (A) and IL-10 (B) levels, respectively. After 48 h, culture supernatants were collected, and IFN-γ and IL-10 levels were analyzed using ELISA. Bars show the mean of three independent experiments. Circles, triangles, and squares represent different batches of fermented *B. rapa* L. preparations. Data were analyzed using a one-way ANOVA followed by Tukey’s multiple comparison test. The letters indicate significant differences (*P*<0.05).

To assess the changes induced in IFN-γ and IL-10 production by fermented *B. rapa* L., mouse spleen cells were stimulated with LS from non-fermented or fermented *B. rapa* L. for 48 h, and the concentrations of IFN-γ and IL-10 in the supernatants were analyzed. Fig. 3A shows the change in IFN-γ production during the fermentation of *B. rapa* L. LS increased IFN-γ production significantly more than non-fermented vegetables on day 7 or 14 after the start of fermentation. IL-10 levels induced by LS were higher on day 14 than those with non-fermented *B. rapa* L., which were lower than IFN-γ levels (Fig. 3B).

### Relationship between cytokine production and LAB numbers

**Fig. 4:**
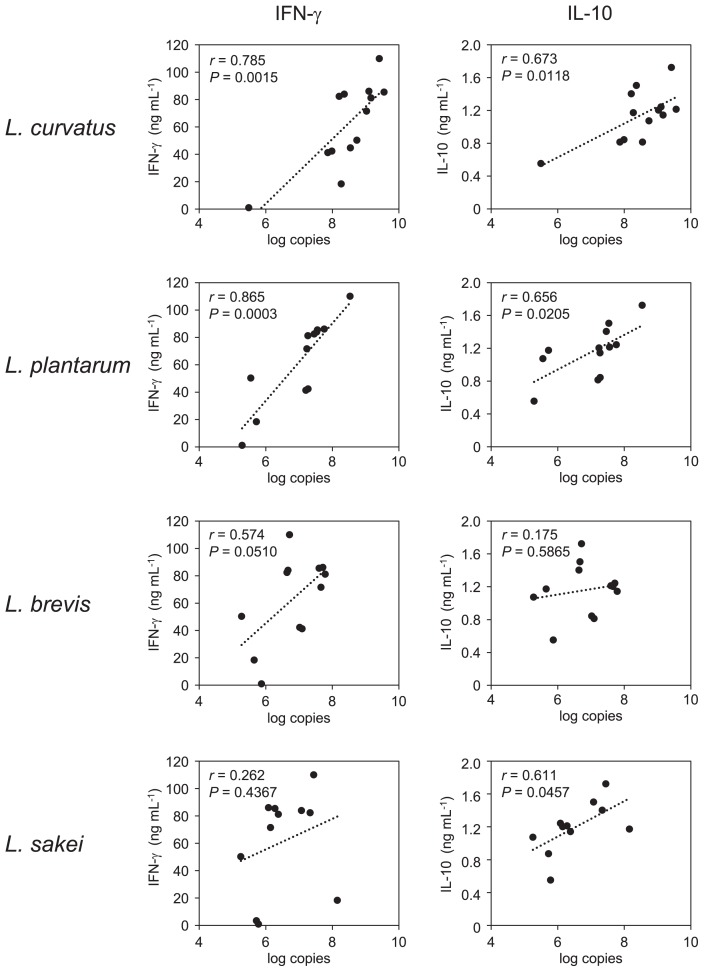
Relationship between cytokine production and the number of LAB. Pearson’s correlation analysis was used to evaluate the relationship between cytokine production and the number of LAB. Each plot shows the number of LAB and cytokine production of all LS from three batches. The detection limits of the number of bacteria were 4 log copies g^–1^, and data below the detection limit were eliminated.

To assess the involvement of each *Lactobacillus* species in the ability of fermented *B. rapa* L. to induce cytokine production, we examined the relationship between cytokine production and the number of each *Lactobacillus* species. The levels of IFN-γ strongly correlated with the numbers of *L. plantarum* (r=0.865; *P*=0.0003) and *L. curvatus* (r=0.785; *P*=0.0015), but not with those of *L. brevis* (r=0.574; *P*=0.0510) and *L. sakei* (r=0.262; *P*=0.4367). *L. curvatus* had the highest correlation coefficient associated with IL-10 production (r=0.673; *P*=0.0118) (Fig. 4).

### Cytokine production by mouse spleen cells treated with LAB strains isolated from fermented *B. rapa* L

**Fig. 5:**
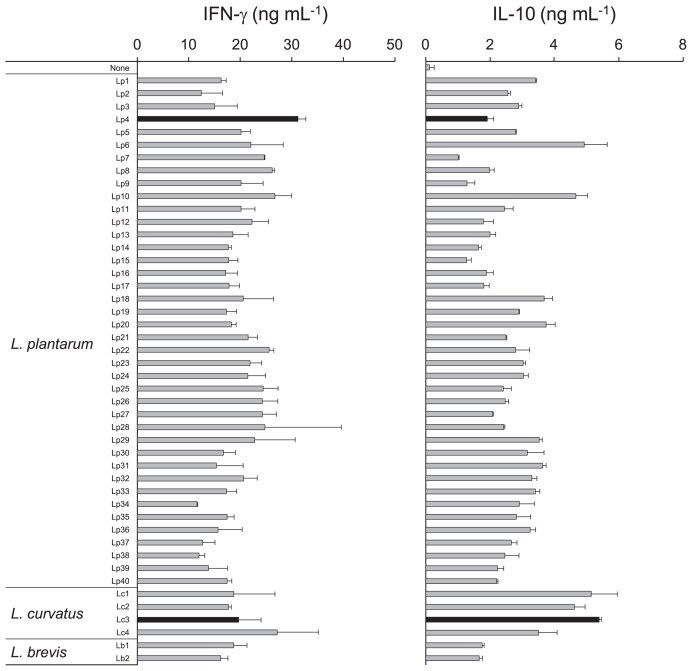
Effects of LAB isolated from fermented *B. rapa* L. on cytokine production by mouse spleen cells. Mouse spleen cells (5×10^5^ cells per well) were treated with 1 and 10 μg mL^–1^ of heat-killed bacteria to measure IFN-γ and IL-10 levels, respectively. After 48 h of culture, cell-free supernatants were collected, and IFN-γ and IL-10 levels were analyzed using ELISA. Data are shown as the mean±SD. Results represent seven independent experiments.

Among 46 isolated LAB strains, 40 were *L. plantarum*, four were *L. curvatus*, and two were *L. brevis*. Although all isolated strains induced IFN-γ and IL-10 production by mouse spleen cells, each strain showed different patterns (Fig. 5). Among 46 strains, Lp4 and Lc3 induced the highest levels of IFN-γ and IL-10, respectively. Therefore, we selected these two strains as starter cultures to produce fermented *B. rapa* L. with the potential to exert immunomodulatory effects.

### Effects of starter cultures on the cytokine-inducing activity of fermented *B. rapa* L

**Fig. 6:**
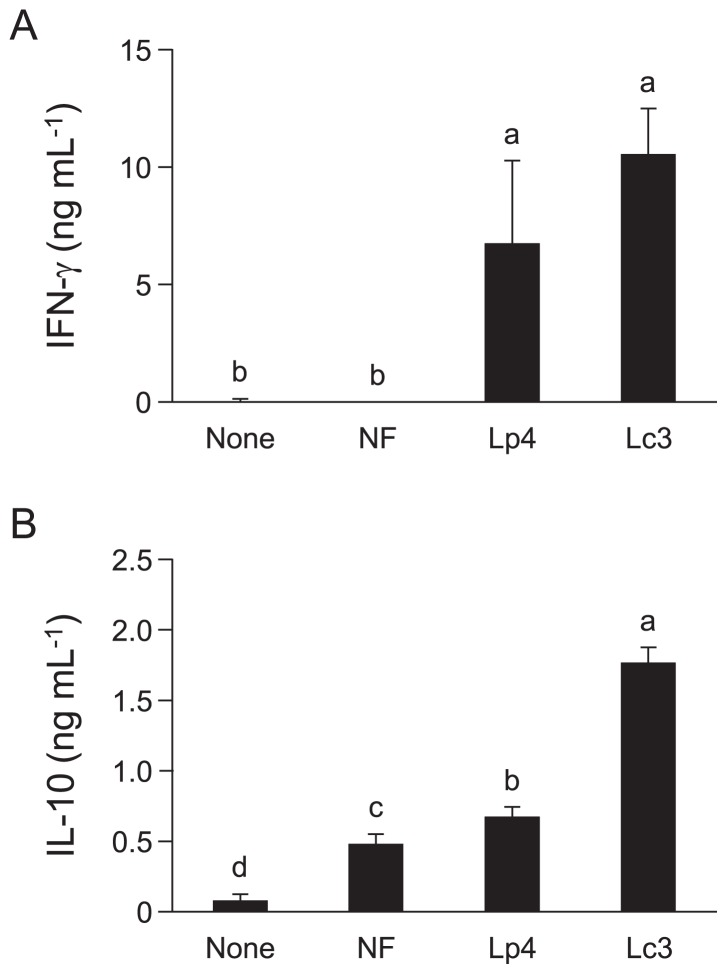
Effects on cytokine production of *B. rapa* L. fermented using starter cultures. *B. rapa* L. fermentations were performed with and without starter cultures (Lp4 or Lc3) at 10°C for 3 d. LS was prepared from each sample. Mouse spleen cells (5×10^5^ cells per well) were stimulated with 0.25 and 10 μg mL^–1^ of LS to measure IFN-γ (A) and IL-10 (B) levels, respectively. After 48 h of culture, cell-free supernatants were collected, and IFN-γ and IL-10 levels were analyzed using ELISA. Results represent two independent experiments. Data are shown as the mean±SD. Statistical analyses were performed as described in Fig. 3. The letters indicate significant differences (*P*<0.05).

To assess the effects on cytokine production of the addition of Lp4 and Lc3 isolates as starter cultures to the fermentation of *B. rapa* L., mouse spleen cell suspensions were treated with LS after 3 d of fermentation, and the concentrations of IFN-γ and IL-10 in the supernatants were measured. IFN-γ was not detectably induced by LS from NF. LS from fermentations of *B. rapa* L. initiated with Lp4 and Lc3 significantly increased the levels of IFN-γ (Fig. 6A). IL-10 production was induced by fermented *B. rapa* L. with starter cultures significantly more than by naturally fermented *B. rapa* L., and Lc3-fermented *B. rapa* L. induced the highest amount of IL-10 produced (Fig. 6B). A quantitative PCR analysis revealed that the addition of starter cultures markedly increased the number of lactobacilli in LS from 3-d fermented *B. rapa* L.; the numbers (log) of lactobacillus 16S sequences derived from the LS of NF, Lp4, and Lc3 were 5.7, 9.2, and 8.9 g^–1^ of fermented *B. rapa* L., respectively. These data were reproducible.

## Discussion

We herein clarified changes in the overall bacterial community during the fermentation of *B. rapa* L., and demonstrated that the number of LAB and cytokine-inducing activity increased during fermentation. The number of LAB correlated with the levels of IFN-γ and IL-10. LAB strains isolated from fermented *B. rapa* L. promoted IFN-γ and IL-10 production by mouse spleen cells. Furthermore, fermented *B. rapa* L. initiated with cultures of *Lactobacillus* isolates increased IFN-γ and IL-10 production significantly more than naturally fermented *B. rapa* L.

An amplicon analysis of the V3–V4 regions of the 16S rRNA gene revealed microbial succession during the fermentation of *B. rapa* L. *Rhizobiales*, *Sphingomonadales*, and *Pseudomonadales* were the most prominent in non-fermented or 3-d fermented vegetables (Table 1). The relative abundance of *Lactobacillales* markedly increased on day 7 of fermentation, and *Lactobacillales* was the most dominant when *B. rapa* L. was fermented for at least 7 d (Table 1). Parameters that represent the diversity of the bacterial community were lower on day 7 than those of non-fermented vegetables (Table 2). These results suggest that increasing the population of *Lactobacillales* produced organic acids that lowered the pH of the brine and vegetables, resulting in a decrease in the populations of other bacterial species. The pH values of 7-d or longer fermented vegetables were significantly lower than that of non-fermented vegetables (data not shown). Similar bacterial succession has been observed during kimchi fermentation, which is mainly mediated by *Lactobacillaceae* (13).

A hierarchical clustering analysis using a weighted Unifrac distance matrix indicated that non-fermented and 3-d fermented vegetables harbored similar bacterial communities, which were distinct from the bacterial communities observed in ≥7-d fermented vegetables (Fig. 1). This suggests that the bacterial community became robust when *B. rapa* L. was fermented for at least 7 d. Thus, the robustness of the microbiota may contribute to elongating the shelf life of pickled vegetables.

A microbial community analysis revealed that the representative sequences of the four most dominant OTUs showed the highest sequence identities with the 16S rRNA genes of *L. curvatus*, *L. plantarum*, *L. sakei*, and *L. brevis*, suggesting that these are major *Lactobacillus* species that are the primary contributors to the fermentation of *B. rapa* L. The numbers of these *Lactobacillus* species throughout fermentations were quantified by real-time PCR using species-specific primers. Real-time PCR analyses revealed that *L. curvatus* was the most dominant lactobacillus, followed by *L. plantarum* and *L. brevis*. In contrast, the numbers of *L. sakei*, which widely varied among batches, were markedly lower. The API50CHL test detects the LAB species *L. curvatus*, *L. plantarum*, *L. brevis*, *L. coprophilus*, *L. delbruekii*, *L. fermentum*, and *Leuconostoc mesenteroides* in fermented *B. rapa* L (15). Some of these species were detected here in fermented *B. rapa* L., suggesting that *B. rapa* L. induced the proliferation of specific LAB species during fermentation.

Non-fermented *B. rapa* L. induced a negligible amount of IFN-γ from mouse spleen cells (Fig. 3A). The levels of IFN-γ increased in fermented *B. rapa* L. after 7 or 14 d of fermentation (Fig. 3A), which corresponds to the times when the number of major *Lactobacillus* species increased (Fig. 2). Mouse spleen cells were treated with LS, which was prepared by the centrifugation of a PBS suspension of non-fermented or fermented vegetables. Thus, LS mainly comprised bacterial cells. Furthermore, the levels of IFN-γ correlated with the numbers of *L. curvatus* and *L. plantarum* (Fig. 4). These two LAB species promote IFN-γ production by mouse spleen cells (32). The cell wall components of LAB, such as peptidoglycan (PGN) and lipoteichoic acid (LTA), induce IL-12 production by murine macrophages and splenic DCs in a TLR2-dependent manner (11, 21). Since LS consisted of not only lactobacilli, but also many types of bacteria, synergistic effects on cytokine production by *Lactobacillus* and other bacteria including *Lactobacillus* are possible. IL-10 production, unlike that of IFN-γ, was relatively high in the LS of non-fermented *B. rapa* L. (Fig. 3B), suggesting that IL-10 was induced by bacteria other than LAB in non-fermented *B. rapa* L. After fermentation, the levels of

IL-10 correlated with the number of LAB species, particularly those of *L. curvatus*, *L. plantarum*, and *L. sakei*. For example, *L. plantarum* strains potently induce IL-10 production by mouse peritoneal macrophages, and the IL-10-induced synthesis of teichoic acids by this strain is mediated by TLR2-dependent ERK activation (14). Therefore, the cell wall component of LAB may induce IL-10 production. The number of *L. sakei* correlated with IL-10 production, but not with IFN-γ production, suggesting that *L. sakei* in fermented *B. rapa* L. may have a stronger IL-10-producing ability than IFN-γ. However, we failed to obtain *L. sakei* isolates, possibly because of weaker dominance.

Fermented *B. rapa* L. induced greater increases in IFN-γ and IL-10 production than non-fermented vegetables, and, thus, lactobacilli appear to be involved in cytokine production. IL-12 and IFN-γ suppress the differentiation of Th2 cells, and IL-10 is a potent inhibitor of inflammation through its suppression of the synthesis of Th2 cytokines (7). Allergic inflammation is characterized by the infiltration of tissues by mast cells and activated eosinophils, which release Th2 cytokines, particularly IL-4 and IL-5 (24). Thus, fermented *B. rapa* L. may suppress allergic reactions through the production of IFN-γ and IL-10 by immune cells. A previous study reported that *L. casei* variety *rhamnosus* alleviated atopic dermatitis, possibly by improving the Th1/Th2 balance and intestinal microbiota (38). Therefore, the ingestion of Lp4 or Lc3 strains may improve the Th1/Th2 balance by changing the gut microbiota.

We demonstrated that the microbiota and immunomodulatory activity were changed by the fermentation of *B. rapa* L. The characteristic of fermentation of *B. rapa* L. is an increase in *L. curvatus*. A previous study demonstrated that *L. mesenteroides* and *L. sakei* were predominantly observed in the early and late fermentation stages of kimchi (6). To the best of our knowledge, this is the first study to investigate the immunomodulatory effects of fermented vegetables.

In the present study, most LAB isolates from *B. rapa* L. fermented for 28 d were identified as *L. plantarum* (Fig. 5). These results are consistent with a previous study demonstrating that *L. plantarum* plays a dominant role in community dynamics during the final phase of the spontaneous fermentation of cauliflower and mixed vegetables (green tomatoes, carrots, and cauliflower) (36). However, a quantitative PCR analysis using species-specific primers revealed that *L. curvatus* was the most dominant lactobacillus (Fig. 2). This difference may be explained by evidence indicating that viable *L. plantarum* are present during the later stages of the fermentation of *B. rapa* L., in contrast to other species. Another possibility is differences in the growth of lactobacilli on MRS agar.

Each LAB strain exhibited differences in the production of IFN-γ and IL-10, although some strains represented the same species (Fig. 5). Certain *L. plantarum* strains induce higher levels of IL-12p70, which is strain-specific rather than species-specific. The strain-dependent induction of cytokine production is explained, in part, by the amount of bacterial PGN (29). Furthermore, the content of LTA in the cell wall of *L. plantarum* differs among strains, which affects IL-12p40 production (11). Therefore, the different cytokine-inducing activities of isolated LAB species from fermented *B. rapa* L. may be explained by their contents of PGN and LTA.

*B. rapa* L. fermentations initiated with starter cultures increased the levels of IFN-γ and IL-10 induction significantly more than with naturally fermented *B. rapa* L. Furthermore, higher numbers of lactobacilli were present in starter-fermented *B. rapa* L. than in natural fermentation. Thus, the addition of starter cultures appears to have induced an early increase in the number of lactobacilli, leading to enhanced cytokine production. However, the level of IL-10 production was markedly higher in Lc3-fermented *B. rapa* L. Fig. 5 shows that the Lc3 strain was associated with higher levels of IL-10 being produced than the Lp4 strain. Thus, the cytokine-inducing activity of LAB strains may affect the levels of cytokine production in fermentations of *B. rapa* L. initiated using starter cultures. Moreover, the use of these starter cultures may enhance the induction of cytokine production, which contributes to the manufacture of fermented *B. rapa* L. *In vivo* feeding studies are needed to identify the immunoregulatory functions of starter-fermented *B. rapa* L.

In summary, we herein demonstrated that the number of LAB increased during the fermentation of *B. rapa* L. and that *L. curvatus* and *L. plantarum* contributed to the ability of fermented *B. rapa* L. to induce the production of IFN-γ and IL-10. The addition of *Lactobacillus* isolates as starter cultures enhanced cytokine induction by fermented *B. rapa* L. Thus, these LAB may be applied to produce fermented foods with immunoregulatory effects.

## Figures and Tables

**Table 1:** Changes in relative abundance of bacterial orders during the fermentation of *Brassica rapa* L.

Taxonomy Phylum/Order	Fermentation (d)						*P* value

	0	3	7	14	21	28	
*Bacteroidetes*							
*Flavobacteriales*	3.05±1.94^a^	3.04±2.24^a^	0.21±0.15^a^	0.12±0.07^a^	0.07±0.04^a^	0.08±0.03^a^	0.013
*Firmicutes*							
*Lactobacillales*	2.43±3.85^b^	18.77±26.55^b^	78.71±12.77^a^	85.67±8.88^a^	83.80±8.23^a^	84.03±9.27^a^	<0.001
*Proteobacteria*							
*Rhizobiales*	38.22±13.72^a^	31.75±14.10^a^	5.23±3.30^b^	3.23±1.05^b^	2.36±0.17^b^	2.07±0.21^b^	0.0002
*Rickettsiales*	4.40±2.15^a^	2.08±0.99^ab^	0.35±0.06^b^	0.29±0.10^b^	0.46±0.2^b^	0.36±0.23^b^	0.0011
*Sphingomonadales*	18.43±4.14^a^	20.44±7.34^a^	3.63±1.81^b^	2.55±1.21^b^	1.55±0.19^b^	2.11±1.22^b^	<0.001
*Burkholderiales*	4.44±1.70^ab^	6.85±4.71^a^	1.21±0.81^ab^	0.63±0.46^b^	0.38±0.19^b^	0.59±0.45^b^	0.0113
*Enterobacteriales*	4.76±6.35^a^	3.64±3.24^a^	8.12±9.99^a^	6.21±7.95^a^	5.03±5.05^a^	5.38±6.62^a^	0.977
*Oceanospirillales*	0±0^a^	0.10±0.16^a^	0.04±0.07^a^	0.07±0.11^a^	1.72±2.98^a^	1.87±3.23^a^	0.599
*Pseudomonadales*	16.66±8.80^a^	8.29±6.82^ab^	1.70±1.55^b^	0.65±0.32^b^	0.29±0.13^b^	0.20±0.11^b^	0.0044
*Vibrionales*	0±0^a^	0.09±0.16^a^	0.04±0.07^a^	0.17±0.29^a^	4.03±6.98^a^	3.02±5.24^a^	0.586
Others	7.63±1.39^a^	4.95±1.95^a^	0.75±0.32^b^	0.41± 0.09^b^	0.29±0.08^b^	0.30±0.14^b^	<0.001

Values are the relative abundance (%) of each order in fermented or non-fermented *Brassica rapa* L., and are shown as the means±SD of percentages of triplicate fermentations. Values with different letters significantly differ (*P*<0.05, Tukey’s test). Others are composed of orders each showing a relative abundance of less than 5% of the total read in all samples.

**Table 2:** Changes in alpha diversity parameters during the fermentation of *Brassica rapa* L.

Parameter	Fermentation (d)						*P* value

	0	3	7	14	21	28	
chao1	945.2±233.6^a^	806.5±103.0^ab^	548.0±101.3^b^	499.3±119.5^b^	484.8±104.8^b^	503.6±158.1^b^	0.0075
observed_otus	626.2±86.2^a^	522.7±116.1^a^	315.8±61.5^b^	278.2±54.7^b^	252.8±41.6^b^	257.9±41.1^b^	<0.001
PD_whole_tree	24.37±7.14^a^	19.45±2.71^ab^	12.52±1.16^bc^	10.75±0.34^bc^	10.36±0.67^c^	10.33±0.47^c^	<0.001
Shannon	5.65±0.39^a^	5.35±0.58^a^	3.17±1.11^b^	2.95±1.16^b^	3.13±0.39^b^	3.12±0.28^b^	0.0012

Values are the means±SD of triplicate fermentations. Values with different letters significantly differ (*P*<0.05, Tukey’s test).
